# Chinese Traditional Medicine NiuBeiXiaoHe (NBXH) Extracts Have the Function of Antituberculosis and Immune Recovery in BALB/c Mice

**DOI:** 10.1155/2021/6234560

**Published:** 2021-01-18

**Authors:** Yan Liang, Wenping Gong, Xiaomei Wang, Junxian Zhang, Yanbo Ling, Jinying Song, Lan Wang, Xiao Liu, Jie Wang, Yourong Yang, Shibing Chen, Jun Liu, Chunwei Yang, Huafeng Luo, Xueqiong Wu

**Affiliations:** ^1^Army Tuberculosis Prevention and Control Key Laboratory/Beijing Key Laboratory of New Techniques of Tuberculosis Diagnosis and Treatment, Institute of Tuberculosis Research, The 8th Medical Centre of Chinese PLA General Hospital, Beijing 100091, China; ^2^Guangdong Qifang Pharmaceutical Co., Ltd., Guangzhou 510075, China

## Abstract

**Background:**

The Traditional Chinese Medicine NiuBeiXiaoHe (NBXH) is a valid antituberculosis (TB) prescription from the experience of clinical practice. However, the mechanism of NBXH extracts' immunotherapy has been poorly understood. Herein, the immunotherapeutic efficacy and the differentially expressed (DE) genes of NBXH extracts were evaluated and identified in BALB/c mice.

**Methods:**

The total RNA was extracted from peripheral blood mononuclear cells, and the DE genes were identified by gene chip. The enrichment and signaling pathway analyses were performed using Gene Ontology (GO) and KEGG database.

**Results:**

It was shown that the treatment of NBXH extracts (high dose) significantly reduced mycobacteria loads and histopathological lesions in mice infected by *Mycobacterium tuberculosis* and resulted in 3,454 DE upregulated genes and 3,594 downregulated DE genes. Furthermore, NBXH extracts killed mycobacteria by inhibiting the supply of necessary ingredients for their growth and proliferation. They restored the disordered immune microenvironments by up- or downregulating immune and inflammation-related pathways.

**Conclusions:**

Taken together, NBXH extracts not only efficiently decreased the mycobacteria loads but also balanced the immune disorders in mice. These new findings provide a fresh perspective for elucidating the immunotherapeutic mechanism of NBXH extracts and pointed out the direction for improving the treatment efficacy of NBXH extracts.

## 1. Introduction

Tuberculosis (TB) is a global contagious disease that can affect almost any part of the body but is mainly found in the lungs. According to the Global TB report 2020 by the World Health Organization, it is estimated that there were 10.0 million TB patients in the world in 2019 [1]. TB is still a serious and growing public health problem. At present, TB is mainly treated with combination chemotherapy of western medicine. However, the treatment of western medicine has the following issues: First, the treatment of multidrug-resistant (MDR) TB and incredibly extensively drug-resistant (XDR) TB is complicated in the clinic, and requires combining second-line drugs and more than nine months of treatment course [2]. Second, antituberculosis chemical drugs usually have side effects; for example, severe liver injury was induced in about 10% of TB patients [3]. Third, the western medicine treatment on TB mainly emphasizes killing *M. tuberculosis* [4].

TB is not only a bacterium infectious disease but also an immune illness. TB's occurrence and development are closely related to immune dysfunction such as abnormal monocyte-macrophage activation, Th1 immune response, the imbalance of Th1/Th2 immune response, and hypoimmunity [5]. Traditional Chinese Medicine (TCM) compound has multicomponent, multitarget, multipathway, and multieffect characteristics. It plays a synergistic effect with chemotherapy on killing *M. tuberculosis*, enhancing the patient's immune function, improving clinical symptoms and sputum negative rate, and increasing the curative percentage of TB [6]. Therefore, the treatment of TB with integrated traditional Chinese and western medicine is likely to open an effective way to extricate TB (especially MDR-TB and refractory TB) from a challenging situation.

**Figure 1:**
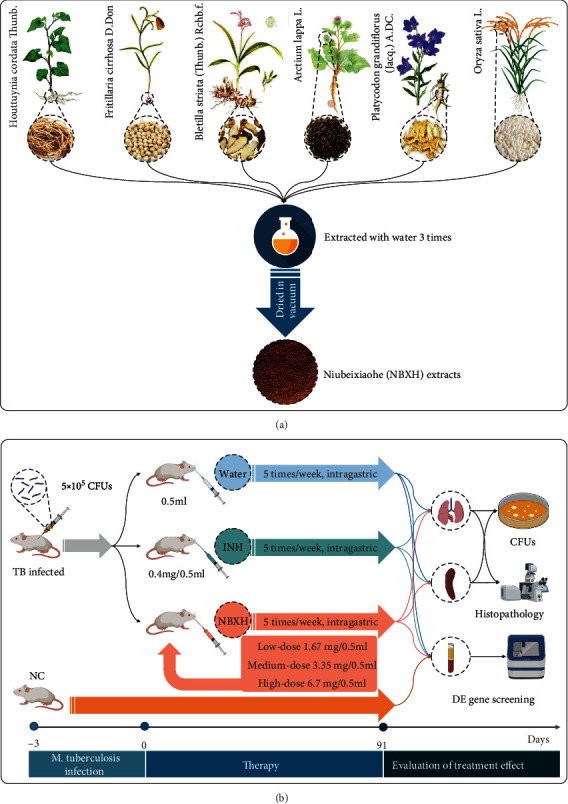
The elements of traditional Chinese medicine NBXH and its flow chart of immunotherapy in a mice model. (a) Schematic diagram of the six natural plants of NBXH. (b) Three days before immunotherapy, 53 female BALB/c mice were infected by *M. tuberculosis* H37Rv strain with 5 × 10^5^ CFUs per mouse. Then, three mice were sacrificed to determine whether the infection model had been successfully constructed. The remaining mice were randomly assigned to five groups and treated with water (negative control); INH (positive control); and NBXH-L, NBXH-M, or NBXH-H by intragastric administration five times in one week. In addition, as a blank control, ten normal mice without any treatment were kept in the same condition. After 91 days of initial immunotherapy, mice were sacrificed, and their lungs, livers, spleens, and blood were collected to evaluate the effect of immunotherapy or identify GE genes.

The TCM compound NiuBeiXiaoHe (NBXH) is a valid anti-TB prescription from clinical practice experience [7]. NBXH is composed of six Chinese herbal medicines (Table 1 and Figure 1(a)). *Bulbus Fritillariae Cirrhosae* and *Rhizoma Bletillae* are the main components of NBXH, which have the functions of heat clearing, lung moistening, phlegm resolving, cough relieving, hemostasis, and detumescence [8–10]. *Herba Houttuyniae*, *Radix Platycodonis*, and *Fructus Arctii* are the auxiliary elements of NBXH, which have the beneficial effects of acting as an anti-inflammatory, reducing phlegm, and relieving cough as well as sore throat [11–13]. Glutinous rice is used as the adjuvant to invigorate the spleen, stomach, and lung [14]. The compound NBXH has been used to treat TB patients in the clinic for more than 20 years, and it could quickly eliminate symptoms such as chest pain, hemoptysis, low fever, night sweat, malnutrition, and fatigue; it could also improve the cure rate of TB [15]. Our previous studies found that NBXH extracts have some therapeutic effects on the TB mouse model by inhibiting or killing *M. tuberculosis in vitro* and *in vivo*, reducing lung lesions, and improving the general condition of mice [16, 17].

However, the immunotherapeutic mechanism of NBXH extracts remains unclear. It was well known that isoniazid (INH) is the first-line drug for TB treatment in the clinic, and hence we chose it for positive control. Herein, we will compare the efficacy of NBXH extracts and INH in the treatment of tuberculosis, identify differentially expressed (DE) genes, and analyze potential target molecules and signaling pathways through gene chip technology and bioinformatics methods to explore the immunotherapeutic mechanism of NBXH extracts.

## 2. Materials and Methods

### 2.1. Mice and Ethics Statement

Female BALB/c mice aged 6-8 weeks (17-19 g) were obtained from Vital River Laboratories (Beijing, China). All animal experiments were approved and directed by the Animal Ethical Committee of the 8th Medical Center of Chinese PLA General Hospital. Animal care and management were strictly carried out under the standards of Experimental Animal Regulation Ordinances formulated by the China National Science and Technology Commission. At the end of the experiments, the mice were euthanized with ketamine hydrochloride (2 mg/kg) and 2% xylazine (3 mg/kg) and then euthanized with cervical dislocation.

### 2.2. *Mycobacterium tuberculosis* Strain

*Mycobacterium tuberculosis* (H37Rv strain) was obtained from the Chinese Academy for Food and Drug Control and cultured on the Lowenstein-Jensen medium. Four weeks later, the mycobacterial colonies were converged to homogenize in saline (0.05% Tween 80) and then stored at -20°C. The colony-forming units (CFUs) of viable mycobacteria were determined by plating serial dilutions on the Lowenstein-Jensen medium.

### 2.3. The Preparation of NBXH Extracts

The Chinese medicine compound NBXH was purchased from Guangdong Qifang Pharmaceutical Co., Ltd. (Guangzhou, China). The weight of each plant was listed in Table 1. The NBXH was extracted with water three times, and their filtrates were combined, concentrated, and then dried in a vacuum. All these steps were achieved by Guangdong Qifang Pharmaceutical Co., Ltd. The details of the NBXH extraction process can be found in our previous study [16].

### 2.4. The Preparation and Treatments of the TB-Infected Mouse Model

A flowchart of the preparation and treatments of the TB-infected mouse model is shown in Figure 1(b). Fifty-three specific pathogen-free female BALB/c mice were challenged with 5 × 10^5^ CFUs of *M. tuberculosis* strain H37Rv suspension via tail vein injection. Three days after the challenge, three mice were randomly selected and sacrificed to determine whether the TB-infected mouse model was successfully constructed. Then, the remaining mice were divided into five groups (10 mice each group) and given distilled Water, INH (Shenyang Hongqi Pharmaceutical Co., Ltd., Liaoning, China), low-dose NBXH extracts (abbreviated as NBXH-L), medium-dose NBXH extracts (abbreviated as NBXH-M), or high-dose NBXH extracts (abbreviated as NBXH-H) by intragastric administration five times per week (except Saturday and Sunday), respectively. The experimental groups were briefly described as follows: negative control—TB-infected mice were treated with 0.5 ml distilled water; positive control—TB-infected mice were treated with 0.4 mg/0.5 ml INH; NBXH-L group—TB-infected mice were treated with 1.67 mg/0.5 ml NBXH extracts; NBXH-M group—TB-infected mice were treated with 3.35 mg/0.5 ml NBXH extracts; and NBXH-H group—TB-infected mice were treated with 6.7 mg/0.5 ml NBXH extracts. The dosage of NBXH extracts used by each mouse was calculated based on the practical clinical dose. Besides, the other ten healthy mice were left untreated and used as normal control (NC) for the DE gene identification. After thirteen weeks of the treatment, mice in the Water, INH, and NBXH groups were sacrificed, and the lung, liver, and spleen were collected to evaluate the immunotherapy efficacy. Furthermore, the blood samples were obtained and used to extract total RNA.

### 2.5. Evaluation of Immunotherapy Efficacy of the NBXH Extracts in TB Infected Mice

The weight changes of mice were recorded once a week since day zero in Figure 1(b). Ninety-one days past the first treatment, the body weight of each mouse was accurately weighed. Then, all mice were sacrificed. Their lungs and spleens were collected to observe gross pathological lesions (the evaluation criteria are listed in Table 2) and calculate organ coefficients (the ratio of organ weight to body weight). After that, the left lung lobe and the upper half of the spleen were homogenized in 3 ml normal saline, respectively. The tissue suspension was serially diluted at a ratio of 1 : 10, 1 : 100, and 1 : 1000 by using normal saline. Then, 0.1 ml diluted suspension was inoculated in duplicate on the modified Lowenstein-Jensen medium plates containing 0.1 *μ*g/ml amphotericin B (North China Pharmaceutical Co., Ltd., Beijing, China) and incubated at 37°C for four weeks. *M. tuberculosis* colonies in each plate were counted and shown as CFUs per organ. Moreover, the right lung lobe of each mouse was fixed in 10% (vol/vol) neutral formalin and embedded in paraffin. The paraffin-embedded tissue sections were stained with hematoxylin/eosin (H&E) according to our previous study [18]. The histopathologic lesions of lung tissue were observed under a microscope, and the lesion area of the lung was calculated by Image-Pro Plus software (Media Cybernetics, Inc., Rockville, MD, USA).

### 2.6. Extraction, Quantification, and Quality Analysis of Total RNA

Blood samples were collected from mice using an ethylenediaminetetraacetic acid dipotassium (EDTA-K2) anticoagulant tube. The peripheral blood mononuclear cells (PBMCs) were isolated from blood samples following a previous study [19]. Total RNA was extracted from PBMCs using Trizol (Qiagen, Germany) and a Total RNA Extraction Kit (Solarbio Life Science, Beijing, China), and purified using a DP412-RNA clean purification kit (Tiangen Biotech, China) by following the manufacturer's instructions. Total RNA of each sample was quantitated and qualified using Ultraviolet-Visible Spectrophotometer NanoDrop ND-1000 (Thermo Fisher Scientific, USA) and standard denaturing agarose gel electrophoresis.

### 2.7. Gene Expression Analyses

Microarray gene expression analysis was conducted by Kang Cheng Biotechnology Co., Ltd. (Shanghai, China) using an Agilent Array platform. Microarray hybridization was performed based on the manufacturer's standard protocols. Briefly, the purified total RNA was reversely transcribed into fluorescent cDNA following Agilent's Quick Amp Labeling protocol (Version 5.7, Agilent, USA). The Cy3-labeled cDNAs were hybridized with the Whole Mouse Genome Oligo Microarray (4x44K, Agilent, USA), representing more than 39,000 mouse gene transcripts with public domain annotations. The microarray slide was washed and then scanned by the Agilent Scanner G2505C. The obtained microarray images were analyzed using Agilent Feature Extraction software (version 11.0.1.1). Quantile normalization and subsequent data processing were implemented using the GeneSpring GX v11.5.1 software package (Agilent, USA).

### 2.8. DE Gene Identification

The significant DE genes between the NC group and the Water-treated group, and between the Water-treated group and the INH or NBXH extract-treated group, were identified by hierarchical clustering, scatter plot, and volcano plots. The *x*-axis and *y*-axis values in the scatter plot were the averaged normalized signal value of each group (log2 scaled). The hierarchical clustering was performed to show the distinguishable gene expression profiling between samples. Volcano plots were constructed by using fold change values (≥2.0) and *P* values (≤0.05) [20].

### 2.9. GO and Pathway Analyses

Gene Ontology (GO) of significant DE genes between the NC and Water-treated groups, and between the Water-treated and INH or NBXH extract-treated groups, were analyzed using the controlled vocabularies provided by the Gene Ontology project (http://www.geneontology.org). Pathway analysis was performed using the latest KEGG (Kyoto Encyclopedia of Genes and Genomes) database to determine the role these DE genes play in biological pathways. Pathway analysis is a functional analysis mapping genes to KEGG pathways. The *P* value (EASE-score, Fisher-*P* value, or Hypergeometric-*P* value) denotes the significance of the pathway correlated to the conditions. The lower the *P* value, the more significant is the pathway (the recommended *P* value cut-off is 0.05) [21].

### 2.10. Statistical Analyses

Statistical analyses were performed using GraphPad Prism 8 software (San Diego, CA, USA). All the data were expressed as mean ± standard error of the mean (SEM). The significance of difference among groups was evaluated by one-way analysis of variance (ANOVA) or the Kruskal-Wallis test (nonparametric test) according to data normality and homogeneity of variances. The *P* value less than 0.05 indicated statistical significance. Gene expression profiling of mouse was analyzed by fold change. The threshold used to screen up- or downregulated genes was fold change ≥ 2.

## 3. Results

### 3.1. High-Dose NBXH Extracts Significantly Reduced *M. tuberculosis* CFUs

**Figure 2:**
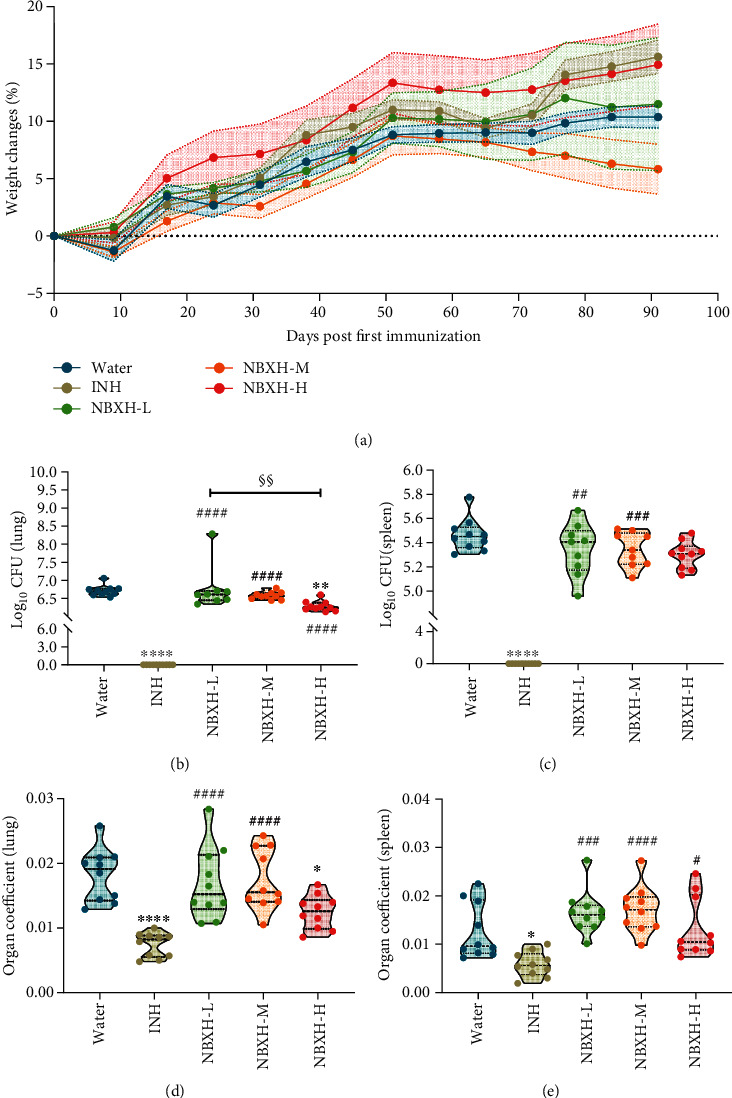
Immunotherapeutic efficacy of NBXH extracts. The original weight of each mouse was obtained on day 0. After the first immunotherapy, the weight change of each mouse was measured weekly (in (a), the error bar is represented by dotted lines). Ninety-one days after initial immunotherapy, all the mice were killed, and their left lobe of the lung (b) or spleen (b) was collected for counting CFUs, and organ coefficient of the lung (d) or spleen (e) was performed. All data are shown as means ± S.E.M. (*n* = 10). Differences were considered statistically significant at *P* < 0.05. ∗*vs.* Water: ^∗^*P* < 0.05; ^∗∗^*P* < 0.01; ^∗∗∗^*P* < 0.001; ^∗∗∗∗^*P* < 0.0001; # *vs.* INH: ^#^*P* < 0.05; ^##^*P* < 0.01; ^###^*P* < 0.001; ^####^*P* < 0.0001; § *vs.* NBXH-H: ^§§^*P* < 0.01.

Three days post infection, three mice were randomly selected to be sacrificed. Spot-like lesions in the lungs and slight enlargement of livers and spleens were observed, and bacillary counts in the lung and spleens were approximately 3.4 log10 and 4.1 log10, which suggested that the TB-infected mouse model had been developed successfully. After thirteen weeks' treatment, the mice in each group were killed, and their organs and blood samples were collected. Our data showed that mice in the NBXH-H or INH treatment group gained 14.93% or 15.62% more weight than initial data, while those in the Water, NBXH-L, or NBXH-M treatment group gained only 10.37%, 11.49%, or 5.83% in weight on 91 days after treatment (Figure 2(a)). Interestingly, we also found that the weight of mice in the NBXH-H or NBXH-L treatment group showed an upward trend from day 0 to day 51, while the weight of mice in other groups showed a decrease on the 9th day after the first immunotherapy.

The mycobacterial loads of organs were evaluated, and the results indicated the following: (1) In the lungs (Figure 2(b)), NBXH-H- (*P* = 0.0088) and INH- (*P* < 0.0001) treated mice had significantly lower CFUs than those in the Water treatment group. INH-treated mice had significantly lower CFUs than those in the NBXH-L (*P* < 0.0001), NBXH-M (*P* < 0.0001), or NBXH-H (*P* < 0.0001) treatment group, and NBXH-H-treated mice had significantly lower CFUs than those in the NBXH-L treatment group (*P* = 0.0044). (2) In the spleens (Figure 2(c)), INH-treated mice had significantly lower CFUs than those in the Water (*P* < 0.0001), NBXH-L (*P* = 0.0030), NBXH-M (*P* = 0.0036), or NBXH-H (*P* = 0.0243) treatment group. Furthermore, the organ coefficient of the organ was performed. The data revealed the following: (1) In the lungs (Figure 2(d)), the organ coefficient of mice treated with INH (*P* < 0.0001) or NBXH-H (*P* = 0.0208) was observably lower than that of mice treated with water, and the organ coefficient of mice treated with INH was observably lower than that of mice treated with NBXH-L (*P* < 0.0001) or NBXH-M (*P* < 0.0001). (2) In the spleens (Figure 2(e)), the organ coefficient of mice treated with INH was lower than that of mice treated with water (*P* = 0.0269), NBXH-L (*P* = 0.0001), NBXH-M (*P* < 0.0001), or NBXH-H (*P* = 0.0136).

### 3.2. High-Dose NBXH Extract-Treated Mice Had Less Histopathological and Gross Pathological Lesions

**Figure 3:**
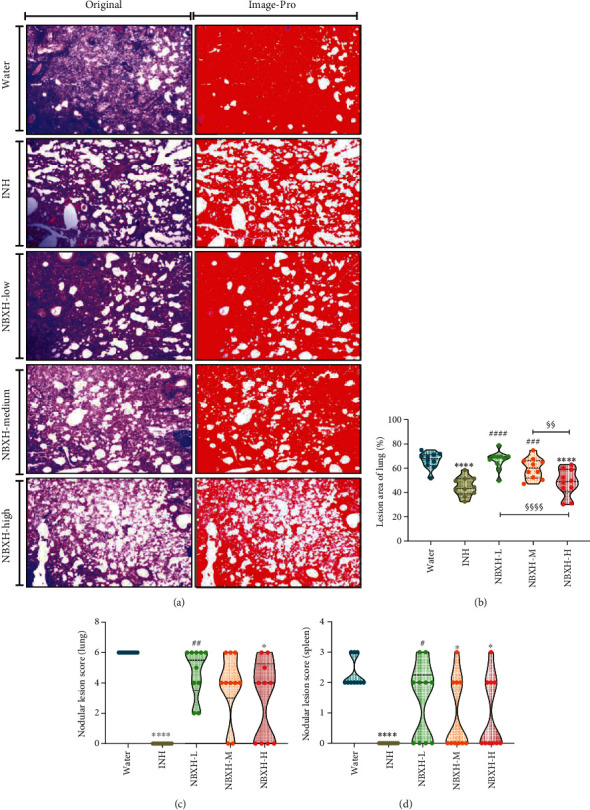
Pathological characteristics of mice treated with NBXH extracts. The right lobe of the lung obtained from the mouse in each group was used to perform the histopathological examination ((a) H&E stain, left lane). The lesion area of the lung was identified as a red color ((a) red, right lane) and analyzed using Image-Pro Plus software (b). Furthermore, the gross pathology of the lung and spleen was also determined, including the nodular lesion score in the lung (c) or spleen (d) according to the evaluation criteria in Table 2. Original magnification times: 40x. All data are shown as means ± S.E.M. (*n* = 10). Differences were considered statistically significant at *P* < 0.05. ∗*vs.* Water: ^∗^*P* < 0.05; ^∗∗^*P* < 0.01; ^∗∗∗^*P* < 0.001; ^∗∗∗∗^*P* < 0.0001; # *vs.* INH: ^##^*P* < 0.01; ^###^*P* < 0.001; ^####^*P* < 0.0001; § *vs.* NBXH-H: ^§§^*P* < 0.01; ^§§§§^*P* < 0.0001.

Histopathological analysis was conducted in the mouse's right lung lobe from each group (Figure 3(a)). Noticeable pathological lesions were observed in mice's lungs in the Water, NBXH-L, or NBXH-M treatment group, such as thickened alveolar walls, a large number of inflammatory cell infiltration, inflammatory exudates in the alveoli, and infiltration of inflammatory cells around the vessel wall. In contrast, the pathological lesions in the lungs of mice treated with INH and NBXH-H were relatively minor, with only a slight thickening of the alveolar wall and a small amount of inflammatory cell infiltration. The lesion area of the lung was evaluated using the Image-Pro Plus software (Figure 3(b)). The results suggested that the lesion area of lungs collected from mice in the INH (*P* < 0.0001) or NBXH-H (*P* < 0.0001) treatment group was smaller than that in the Water treatment group, and the lesion area of lungs collected from mice in the NBXH-L (*P* < 0.0001) or NBXH-M (*P* = 0.0003) treatment group was bigger than that in the INH treatment group. The lesion area of lungs collected from mice in the NBXH-L (*P* < 0.0001) or NBXH-M (*P* = 0.0034) treatment group was significantly bigger than that in the NBXH-H treatment group. Moreover, the gross pathological analysis was also measured. Our data showed the following: (1) In the lungs (Figure 3(c)), the nodular lesion score of mice treated with INH (*P* < 0.0001) and NBXH-H (*P* = 0.0207) was significantly lower than that of mice treated with water, and the nodular lesion score of mice treated with INH was significantly lower than that of mice treated with NBXH-L (*P* = 0.0021). (2) In the spleens (Figure 3(d)), the nodular lesion score of mice treated with INH (*P* < 0.0001), NBXH-M (*P* = 0.0187), or NBXH-H (*P* = 0.0187) was significantly lower than that of mice treated with water, and the nodular lesion score of mice treated with INH was significantly lower than that of mice treated with NBXH-L (*P* = 0.0187).

### 3.3. Identification of DE Genes before and after TB Infection or INH and NBXH-H Treatment

**Figure 4:**
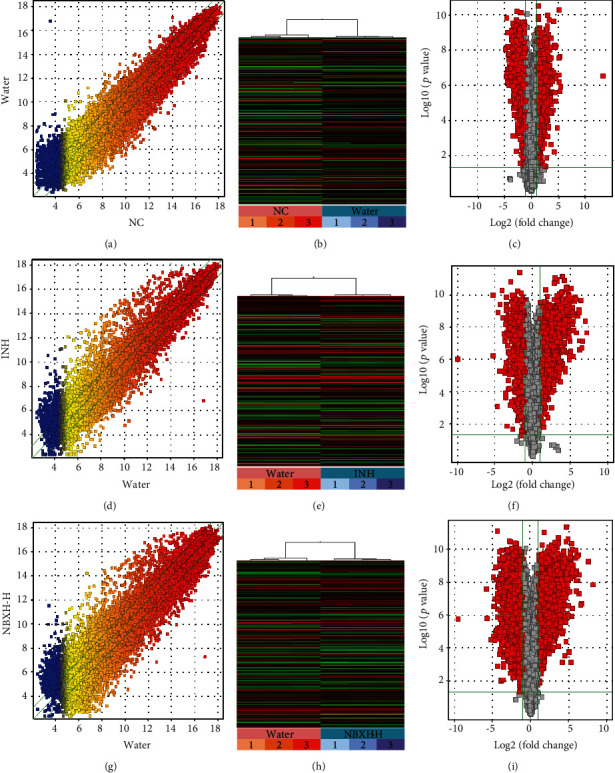
Scatter plot, hierarchical clustering, and volcano plot of the expression profile of genes between Water and control, or INH as well as NBXH-H treated groups. (a, d, and g) Scatter plots; the values of *x* and *y* axes in the scatter plots are the normalized signal values of the samples (log2 scaled) or the averaged normalized signal values of the groups (log2 scaled). The green lines are fold change lines (the default fold change value given is 2.0). The genes above the top green line and below the green bottom line indicated more than a 2-fold change of genes between two samples or groups (*n* = 3). (b, e, and h) Hierarchical clustering shows the relationships among gene expression patterns of samples (*n* = 3). Red indicates high relative expression, and green indicates low relative expression. (c, f, and i) DE genes with statistical significance were identified through the volcano plot, and the red diamonds represent DE genes with fold change ≥ 2.0, *P* ≤ 0.05 (*n* = 3).

Based on the above experimental results, we selected NBXH-H for subsequent screening and analysis of DE genes. The gene expression variation between the two groups was visualized by scatter plots (Figures 4(a), 4(d), and (g)), hierarchical clustering (Figures 4(b), 4(e), and (h)), and volcano plots (Figures 4(c), 4(f), and 4(i)). The results of hierarchical clustering showed a distinguishable gene expression profiling between the groups. The significant DE genes between the two groups were identified through volcano plot filtering (fold change ≥ 2.0, *P* value ≤ 0.05) following our previous study [20]. The results revealed that 2,898 upregulated and 2,749 downregulated DE genes were identified between the Water treatment group and the NC group; 2,111 upregulated and 2,275 downregulated DE genes were identified between the INH treatment group and the Water treatment group; and 3,454 upregulated and 3,594 downregulated DE genes were identified between the NBXH-H treatment group and the Water treatment group, respectively (Table S1).

Surprisingly, we found that 23 genes were significantly downregulated or unchanged in mice infected with *M. tuberculosis*. However, all of them were upregulated considerably after INH or NBXH-H treatment (Table 3). In contrast, 18 genes were significantly upregulated or unchanged in mice infected with *M. tuberculosis*, whereas all were significantly downregulated after INH or NBXH-H treatment (Table 4).

### 3.4. GO Analyses of DE Genes before and after TB Infection or INH and NBXH-H Treatment

**Figure 5:**
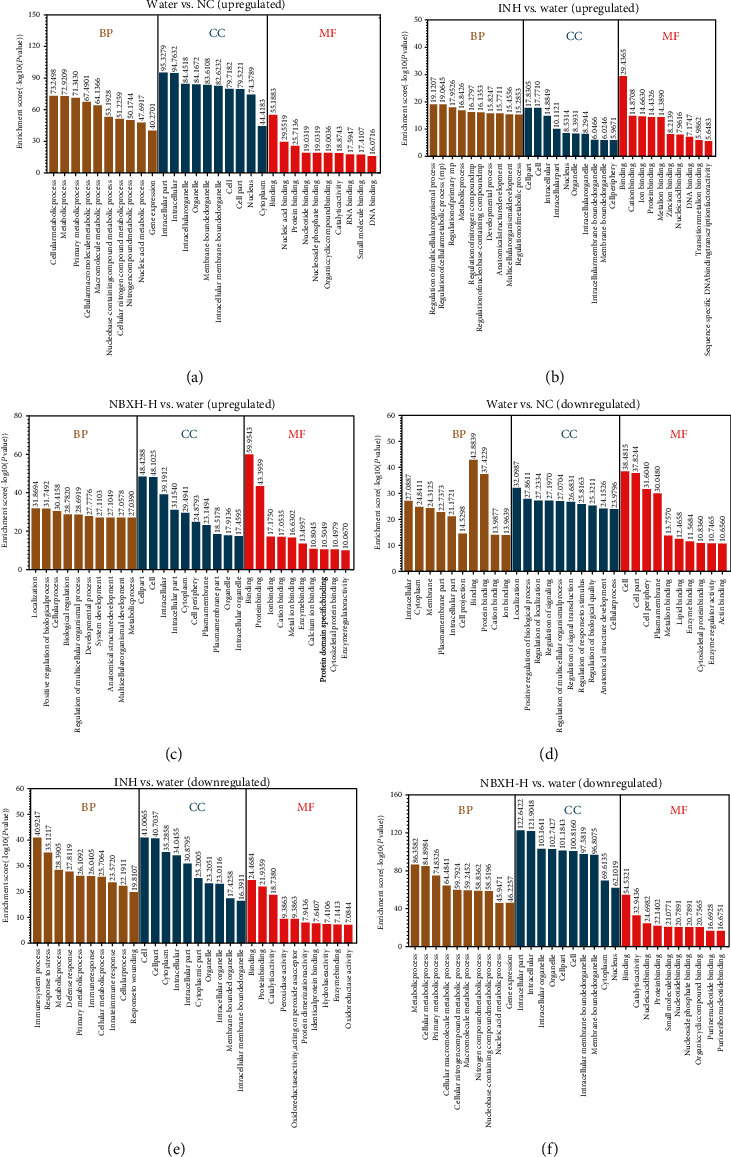
GO analysis of DE genes between before and after *M. tuberculosis* infection and INH or NBXH-H treatment. The top 10 GO terms involved in upregulated DE genes between TB-infected mice and normal mice ((a) Water *vs.* NC), between INH-treated mice and TB-infected mice ((b) INH *vs.* Water), or between NBXH-H-treated mice and TB-infected mice ((c) NBXH-H *vs.* Water) were, respectively, identified in BP, CC, and MF. Similarly, the top 10 GO terms involved in downregulated DE genes between the Water *vs.* NC groups (d), the INH *vs.* Water groups (e), or the NBXH-H *vs.* Water groups (f) were identified in BP, CC, and MF, respectively. The enrichment score is shown as -log10 (*P* value); the *P* value denotes the significance of GO Term enrichment in the DE gene list. The less the *P* value is, the more significant of the GO Term is (*P* ≤ 0.05 is recommended). BP: biological process; CC: cellular component; MF: molecular function.

**Figure 6:**
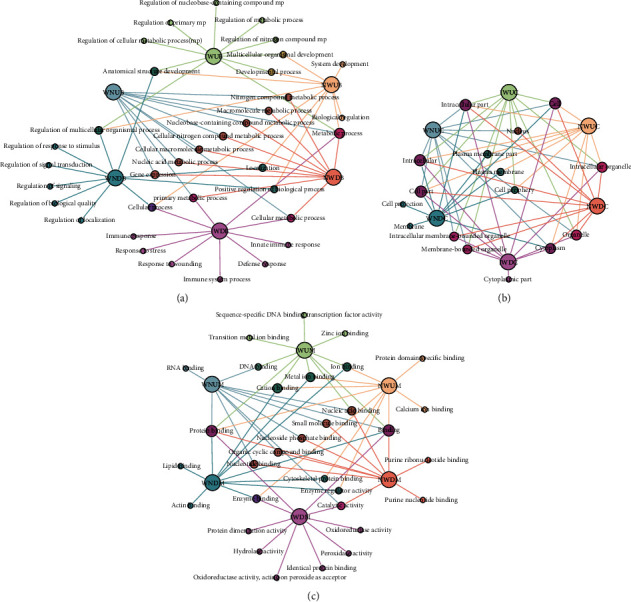
Relationship networks of DE genes between before and after *M. tuberculosis* infection and INH or NBXH-H treatment. The BP (a), CC (b), or MF (c) relationship networks among the normal control, Water, INH, and NBXH-H groups were conducted by Gephi software. WNUB, IWUB, NWUB or WNDB, IWDB, NWDB represented up- or downregulated DE genes involved in BP among the four groups. WNUC, IWUC, NWUC or WNDC, IWDC, NWDC represented up- or downregulated DE genes involved in CC among the four groups. WNUM, IWUM, NWUM or WNDM, IWDM, NWDM represented up- or downregulated DE genes involved in MF among the four groups. Relationships of interest are shown in bold lines.

According to the listed ID of the gene ontology term used in the Gene Ontology Project (GOID), significantly upregulated or downregulated DE genes were identified, respectively, in the biological process (BP), cellular component (CC), and molecular function (MF) by GO analyses. The top 10 GO terms of upregulated or downregulated DE genes sorted by enrichment scores in BP, CC, and MF are shown in Figures 5(a)–5(f), respectively. By analyzing these data, we unexpectedly found the following: (1) In *M. tuberculosis*-infected mice (Water treatment group), seven items in BP were significantly enriched with upregulated DE genes. In contrast, in mice treated with NBXH-H, these items were enriched considerably with downregulated DE genes. These terms included gene expression, nucleic acid metabolic process, cellular macromolecule metabolic process, cellular nitrogen compound metabolic process, nucleobase-containing compound metabolic process, macromolecule metabolic process, and nitrogen compound metabolic process (Figure 6(a)). (2) Three terms in CC were significantly enriched with downregulated DE genes in mice infected with *M. tuberculosis*, while these were enriched with upregulated DE genes in mice treated with NBXH-H. These terms included the plasma membrane part, plasma membrane, and cell periphery (Figure 6(b)). (3) Five terms in MF were significantly enriched with upregulated DE genes in mice infected with *M. tuberculosis*, while these were enriched with downregulated DE genes in mice treated with NBXH-H. These terms included nucleotide binding, organic cyclic compound binding, nucleoside phosphate binding, small ion binding, and nucleic acid binding. Also, five terms in MF were significantly enriched with downregulated DE genes in mice infected with *M. tuberculosis*. In contrast, these were enriched with upregulated DE genes in mice treated with NBXH-H. These terms included cytoskeletal protein binding, enzyme regulator activity, catalytic activity, cation binding, metal ion binding, and ion binding (Figure 6(c)).

Based on the above fact, we focused our analysis on the Water treatment group vs. the NC group and the NBXH-H treatment group vs. the Water treatment group. The top 10 terms of BP, CC, and MF ranked by the enrichment of DE genes are shown in Figure S1. After comparing the data obtained from the TB-infected mice and the NBXH-H-treated mice, we found that the number of upregulated or downregulated genes caused by NBXH-H treatment was more than that caused by TB infection in GO in terms of BP, CC, and MF. Moreover, the upregulated terms of BP, CC, and MF in TB-infected mice were downregulated after NBXH-H treatment, and the downregulated terms of BP, CC, and MF in TB-infected mice were upregulated after NBXH-H treatment.

### 3.5. Pathway Analyses of DE Genes before and after TB Infection or INH and NBXH-H Treatment

**Figure 7:**
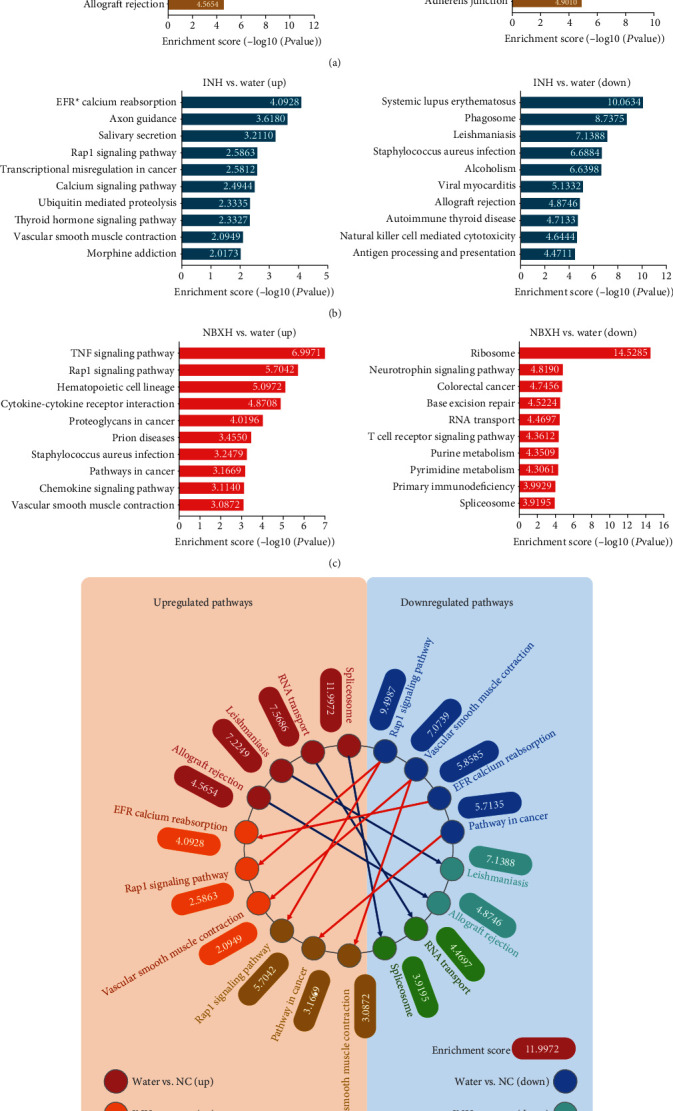
Pathway analysis and relationship networks of DE genes between before and after *M. tuberculosis* infection and INH or NBXH-H treatment. Pathway bar plot explanation and pathway map explanation. The significantly up- or downregulated pathways were ranked by enrichment score (-log10 (*P* value)) value. The top 10 up- or downregulated pathways between the Water-treated group and the NC group (a), between the INH-treated group and the Water-treated group (b), or between the NBXH-H-treated group and the Water-treated group (c) are shown as bar plots. Additionally, the relationship networks of DE genes associated with the top 10 up- or downregulated pathways are made by PowerPoint software (d). The pathways located in the orange region on the left were significantly upregulated, while the pathways located in the light blue zone on the right were significantly downregulated. The interactions between pathways that were significantly upregulated in the Water *vs.* NC groups but downregulated in the INH *vs.* Water or the NBXH-H *vs.* Water groups are shown in the blue lines, the interactions between pathways that were significantly downregulated in the Water *vs.* NC groups but upregulated in the INH *vs.* Water or the NBXH-H *vs.* Water groups are shown in the red lines.

The pathway analysis was carried out to identify the potential biological pathways by using the latest KEGG database. Our results indicated the following: (1) Compared with the NC group, 66 significant upregulated pathways or 78 significant downregulated pathways were identified in the Water treatment group (Table S2), the top 10 of which are shown in Figure 7(a). (2) Compared with the Water treatment group, 30 significant upregulated pathways or 67 significant downregulated pathways were identified in the INH treatment group (Table S2), the top 10 of which are shown in Figure 7(b). (3) Compared with the Water treatment group, 78 significant upregulated pathways or 75 significant downregulated pathways were identified in NBXH-H treatment group (Table S2), the top 10 of which were shown in Figure 7(c). In addition, we also analyzed the distribution of the top 10 signaling pathways in each group of mice (Figure 7(d)). It was found that four pathways (Spliceosome, RNA transport, Leishmaniasis, and allograft rejection) were upregulated in TB-infected mice but were downregulated after INH or NBXH-H treatment. Four pathways (Rap1 signaling pathway, vascular smooth muscle contraction, EFR calcium reabsorption, and pathway in cancer) were downregulated in TB-infected mice. In contrast, they were upregulated after INH or NBXH-H treatment.

**Figure 8:**
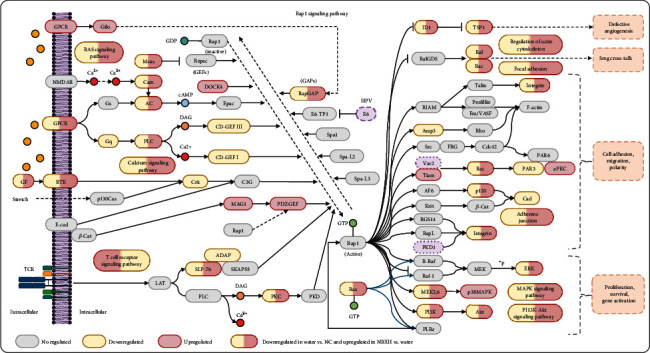
Pathway map explanation of the RAP1 signal pathway. Light yellow marked nodes are associated with downregulated genes; light red marked nodes are associated with upregulated genes; light gray nodes have no significance. The nodes that are light yellow on the left and light red on the right indicate genes or pathways that are downregulated in *M. tuberculosis*-infected mice (Water vs. NC) but upregulated after NBXH-H treatment (NBXH-H *vs.* Water). The nodes that are light red on the left and light yellow on the right indicate genes or pathways that are upregulated in *M. tuberculosis*-infected mice (Water *vs.* NC) but downregulated after NBXH-H treatment (NBXH-H *vs.* Water). ^∗^Endocrine and other examples of factor-regulated calcium reabsorption.

Our results found that the Rap1 signaling pathway plays a vital role in NBXH immunotherapy. Therefore, we focused on this pathway. Rap1 is a small GTPase that plays a significant role in controlling cell-cell and cell-matrix interactions by regulating the functions of integrins and other adhesion molecules in various cell types. As shown in Figure 8, we found that 21 DE genes were significantly downregulated in TB-infected mice but were distinctly upregulated after treatment with NBXH-H, including *GF*, *GPCR*, *RTK*, *M-ras*, *Cam*, *AC*, *PLC*, *SLP-76*, *PKC*, *RapGAP*, *Ras*, *ID1*, *TSP1*, *Ral*, *Rac*, *Integrin*, *p120*, *ERK*, *MEK3,6*, *PI3K*, and *Akt*. Additionally, seven pathways were downregulated in TB-infected mice. Simultaneously, they were upregulated in NBXH-H-treated mice, including the RAS signaling pathway, calcium signaling pathway, regulation of actin cytoskeleton, focal adhesion, adherens junction, MAPK signaling pathway, and PI3K-Akt signaling pathway. In contrast, the T cell receptor signaling pathway was remarkably upregulated in TB-infected mice but was downregulated in NBXH-H-treated mice.

Furthermore, based on the concept that TB is an infectious disease and an immune disease, we compared the upregulated or downregulated pathways related to immune disease and inflammation among different groups (Table 5). Our study found that eight pathways were downregulated after TB infection and upregulated after NBXH-H treatment, including the TNF signaling pathway, regulation of actin cytoskeleton, the Ras signaling pathway, focal adhesion, the PI3K-Akt signaling pathway, the calcium signaling pathway, the MAPK signaling pathway, and the TGF-beta signaling pathway. Four pathways were upregulated after TB infection and downregulated after NBXH-H treatment, including the NF-kappa B signaling pathway, the Toll-like receptor signaling pathway, intestinal immune network for IgA production, and the T cell receptor signaling pathway.

## 4. Discussion

TCM has been practiced in China for thousands of years and has saved countless lives. With the rapid development of modern medicine, a growing number of studies focused on understanding and applying Western medicine methods and techniques to study the pharmacology and efficacy of TCM-derived herbs [22]. As an ancient infectious disease, TB has accompanied humans from the Stone Age till today [23]. TB has been studied for 120 years, but the history of Chinese people using TCM to treat TB can be traced back 500 years ago. To date, several herbal medicines have been demonstrated to have immunotherapeutic efficacies on *M. tuberculosis* infection in animal models, including Allicin [24], Baicalin [25], Yokuinin [26], Mao-Bushi-Saishin-To [26], *Astragalus mongholicus* Bunge [27], *Paeonia lactiflora* Pall. [27], *Curcuma longa L.* [28], and a water extract of *Ranunculus ternatus Thunb.*, *Sophora flavescens Aiton*, *Prunella vulgaris L.*, and *Stellera chamaejasme L.* [29].

In a previous study, we compared the differences between three NBXH extraction methods on a mouse model. We found that the extracts from the three methods all had significant therapeutic effects against TB infection [16]. In the current study, we assessed the immunotherapeutic efficacy of the NBXH extracts (three doses) or INH in TB-infected BALB/c mice. Our results indicated that compared with the mice treated with water, the high-dose NBXH extracts or INH-treated mice gained more weight, had a greater number of CFUs and a significantly lower organ coefficient in the lung, and the pathological lesions of the lungs were lighter. It is noteworthy that INH had a more vital antituberculosis ability than NBXH extracts in both lungs and spleens of mice, indicating that its mechanism of killing intracellular mycobacteria might be different from NBXH extracts. As a prodrug, INH must be activated and catalyzed to the isonicotinic acyl radical by a bacterial catalase-peroxidase enzyme named KatG and coupled with NADH to form the nicotinoy1-NAD complex, which can bind tightly to InhA to inhibit the synthesis of the mycobacterial cell wall [4].

Although INH's action mechanism has been studied relatively clearly, that of NBXH extracts is poorly understood. Based on our previous research, we have proposed the following hypothesis: NBXH extracts would cause differential expression of genes in mice infected with TB. This might provide a new perspective for elucidating the antituberculosis mechanism of NBXH extracts. Encouragingly, 3,454 upregulated and 3,594 downregulated DE genes were identified between high-dose NBXH extracts and the Water treatment groups. Among these DE genes, the top 1 significant upregulated and downregulated DE genes were *4833415N18Rik* (GenBank Accession No: AK014704) and *H2-Oa* (GenBank Accession No: NM_008206), respectively. The former is located on mouse chromosome 16 and is a biased expression in adult frontal lobe, adult cortex, and two other tissues [30], but little research has been done on this gene. In contrast, the latter (also known as *H2-Oa* or *HLA-DO* gene in mice) is a highly conserved nonpolymorphic major histocompatibility complex class II (MHC II) molecule that enhances bacterial antigens' presentation rather than endogenous self-antigens by inhibiting dissociation of class II-associated invariant chain peptides [31]. In addition to the *4833415N18Rik* and *H2-Oa* genes, several other DE genes that have been up- or downregulated in this study have also been reported to be associated with tuberculosis, including *Park2*, *Sp110*, *Dusp4*, and *Klrk1*. The *Park2* gene encodes the protein parkin RBR E3 ubiquitin protein ligase, which is a ubiquitin ligase that plays an essential role in mitochondrial phagocytosis. Previous studies demonstrated that genetic polymorphisms in the *Park2* gene were associated with increased susceptibility to TB [32]. Parkin has a role in ubiquitin-mediated autophagy of *M. tuberculosis* [33]. The *SP110* gene encodes an interferon-induced nuclear protein (Sp110) that plays a crucial role in controlling innate immunity and shaping the inflammatory milieu to *M. tuberculosis* infection [34]. Further study revealed that Sp110 upregulated the apoptotic pathway to enhance host immunity to fight against TB infection via activating endoplasmic reticulum (ER) stress-induced apoptosis [35]. *Dusp4* is the encoding gene of dual-specificity MAP kinase phosphatase 4 (DUSP4) that plays a role in regulating *M. tuberculosis* survival in macrophages and the expression of chemokines [36]. NK cell lectin-like receptor K1 (KLRK1 or NKG2D), encoded by *Klrk1* gene, is an activating receptor on natural killer (NK) and T cells to lyse *M. tuberculosis*-infected monocytes and alveolar macrophages, and a promising target for regulating CD8^+^ T cell-mediated protection against TB [37].

By analyzing the above data, we can draw the following conclusions: after infection with *M. tuberculosis*, mice can activate their immune system to fight the invasion of *M. tuberculosis* by upregulating or downregulating immune- and inflammation-related genes. However, this activation often carries the risk of immune dysfunction named tuberculosis-associated immune reconstitution inflammatory syndrome (TB-IRIS), characterized by an exaggerated inflammatory immune response toward mycobacterial infection [38]. Fortunately, we found that after treatment with NBXH extracts, those exaggerated genes in mice were upregulated or downregulated, and immune balance was restored. Therefore, our research has shown that NBXH extracts could significantly reduce loads of *M. tuberculosis* in the lungs of mice, and more importantly, it played a vital role in the regulation of immune balance. This finding was verified in our subsequent GO analysis and pathway analysis. In GO analysis, it was found that BP terms related to gene expression and metabolic process and MF terms related to nucleotide binding, organic cyclic compound binding, nucleoside phosphate binding, small ion binding, and nucleic acid binding were upregulated in TB-infected mice, but were downregulated after NBXH extract treatment. After *M. tuberculosis* invasion, it is first captured by alveolar macrophages, dendritic cells, and mononuclear cells, but the bacteria can cleverly escape the strangulation of phagosomes to multiply in it. *M*. *tuberculosis* infection usually induces the host cells to enhance the biological processes such as gene expression and metabolism. It improves the ability to obtain the necessary components for mycobacterial cell membrane and nucleic acid synthesis, which provides the conditions required for the survival and proliferation of *M. tuberculosis* [39].

Interestingly, with the continued immunotherapy of NBXH extracts, these favorable conditions created by *M. tuberculosis* for itself was broken, which provided an opportunity for host immune cells to kill mycobacteria and balance immune disorders. This hypothesis was also confirmed in our pathway analysis. Our results suggested that several pathways over downregulated or upregulated by *M. tuberculosis* infection were restored after NBXH extract treatment. These pathways include the Spliceosome, RNA transport, the Rap1 signaling pathway, regulation of actin cytoskeleton, the Ras signaling pathway, focal adhesion, the PI3K-Akt signaling pathway, the calcium signaling pathway, the MAPK signaling pathway, the TGF-beta signaling pathway, the NF-kappa B signaling pathway, the Toll-like receptor signaling pathway, and the T cell receptor signaling pathway. For thousands of years, *M. tuberculosis* has coevolved with the human immune system, which cleverly hijacks the immune mechanism in host cells, making it persistent in infected cells [40]. The long-term survival of mycobacteria depends on their ability to perceive and accommodate the host's hostile conditions. *M. tuberculosis* evades the chase of host immune cells. However, it still can be sensed by the host cells. Therefore, the host will mobilize all available resources to enhance the immune response to release cytokines, proinflammatory cytokines, nitric oxide, and respiratory burst to destroy the favorable conditions for the long-term survival of *M. tuberculosis* [41]. Unfortunately, although the excessively activated or suppressed immune response has played a role in killing mycobacteria, the host has also suffered severe pathological damage. Therefore, it is an ideal immune strategy to enhance mycobacteria eradication and reduce excessive pathological damage [40].

Additionally, there are some shortcomings in this study: (1) The identification of DE genes was conducted in mice rather than human beings, which will increase the uncertainty caused by racial differences. (2) The results from pathway analysis were based on bioinformatics rather than experiments *in vivo* or *in vitro*.

## 5. Conclusion

In summary, this study showed that the Chinese traditional medicine NBXH extracts (high-dose) had a remarkable immunotherapeutic effect, and its treatment resulted in the upregulation of 3,454 DE genes and the downregulation of 3,594 DE genes. Interestingly, our further analysis found that the top-ranked significantly DE genes were mainly enriched in immune and inflammatory responses, and NBXH extracts eliminated the mycobacteria by inhibiting the ingredients required for bacterial growth and proliferation. Furthermore, NBXH extracts revealed an excellent ability in restoring the host's immune microenvironment that was silenced or excessively activated by mycobacterial infection via regulating inflammation and immune-related pathways. Our study found for the first time that high-dose NBXH extracts not only effectively reduced loads of *M. tuberculosis* in the lungs of mice but also played a vital role in balancing host immune disorders caused by *M. tuberculosis* infection. These findings lay the foundation for understanding the immunotherapeutic mechanism of NBXH extracts and provide a way to improve its immunotherapeutic efficacy further.

## Figures and Tables

**Table 1:** The components of NBXH.

Latin name^a^	English name	Authorized names^b^	Chinese name	Plant part	Weight (g)
*Fritillaria cirrhosa* D. Don	*Bulbus Fritillariae Cirrhosae*	*Fritillariae Cirrhosae Bulbus*	Chuan Bei Mu (川贝母)	Bulb	165
*Bletilla striata* (Thunb.) *Rchb*.f.	*Rhizoma Bletillae*	*Bletillae Rhizoma*	Bai Ji (白芨)	Tuber	250
*Houttuynia cordata* Thunb.	*Herba Houttuyniae*	*Houttuyniae Herba*	Yu Xing Cao (鱼腥草)	Stems and leaves	135
*Platycodon grandiflorus* (Jacq.) A. DC.	*Radix Platycodonis*	*Platycodonis Radix*	Jie Geng (桔梗)	Root	165
*Arctium lappa* L.	*Fructus Arctii*	*Arctii Fructus*	Niu Bang Zi (牛蒡子)	Fruits	135
*Oryza sativa* L. var. Glutinosa Matsum	Glutinous rice	No record	Nuo Dao (糯稻)	Polished kernel	150

^a^Full botanical plant names were obtained from The Plant List (http://www.theplantlist.org) or MPNS (http://mpns.kew.org). ^b^The authorized names of traditional Chinese medicine were from the 2015 edition of the Pharmacopeia of the People's Republic of China.

**Table 2:** The evaluation criteria for the gross pathological change of mouse organs.

Organs	Lesion indexes			
	—	+	2+	3+
Lung	Without lesion and necrosis	Lesion number ≤ 10 or necrotic range ≤ 20%	Lesion number > 10, ≤20, or necrotic range > 20%, ≤40%	Lesion number > 20 or necrotic range > 40%
Liver	Normal size	Swell < 20%	Swell ≥ 20%, <40%	Swell ≥ 40%
Spleen	Normal size	Swell < 20%	Swell ≥ 20%, <40%	Swell ≥ 40%

**Table 3:** The significant upregulated genes after NBXH-H treatment.

GenBank accession	Gene name	FCAbsolute^∗^			Gene ontology annotations		
		Water vs. NC	INH vs. Water	NBXH-H vs. Water	Cellular component	Molecular function	Biological process
AK014704	4833415N18Rik	No	62 (↑)	186 (↑)	No	No	No
NM_146630	Olfr123	4 (↓)	73 (↑)	100 (↑)	Membrane	No	Cellular process
NM_001166464	Spock1	9 (↓)	28 (↑)	92 (↑)	Cell part	Binding molecular function	Cellular process
NM_021455	Mlxipl	No	73 (↑)	76 (↑)	Cell part	Binding molecular function	Positive regulation of biological process
NM_010916	Nhlh1	2 (↓)	52 (↑)	72 (↑)	Cell part	Binding molecular function	Cellular process
XR_141090		No	120 (↑)	68 (↑)			
NM_133893	Oas1d	2 (↑)	81 (↑)	67 (↑)	Intracellular	Binding molecular function	Metabolic process, biological regulation
AK087822	Gm7644	No	37 (↑)	63 (↑)	No	No	No
NM_001013362	Npcd	No	34 (↑)	60 (↑)	Cell part	Binding molecular function	No
AK035840	9030624J02Rik	5 (↓)	22 (↑)	58 (↑)	No		
NM_016916	Blcap	No	45 (↑)	57 (↑)	Membrane	No	Cellular process
AK089977	Gm5608	3 (↓)	40 (↑)	49 (↑)	No	No	No
NM_175164	Arhgap26	No	27 (↑)	46 (↑)	Cell part	Binding molecular function	Cellular process
NM_029797	Mnd1	No	No	46 (↑)	Cell part	Binding molecular function	Cellular process
NM_011192	Psme3	No	26 (↑)	46 (↑)	Cell part	Binding molecular function	Cellular process
NM_178227	Scn3b	No	15 (↑)	44 (↑)	Cell part	Binding molecular function	Localization
NM_207017	Tas2r109	5 (↓)	27 (↑)	43 (↑)	Membrane	No	Cellular process
NM_016694	Park2	No	68 (↑)	41 (↑)	Cell part	Binding molecular function	Localization
NM_009299	Sva	No	17 (↑)	41 (↑)	Extracellular space	No	Cellular process
NM_053104	Rbfox2	No	17 (↑)	41 (↑)	Cell part	Binding molecular function	Cellular process
NM_001253361	Kcnma1	5 (↓)	41 (↑)	40 (↑)	Cell part	Binding molecular function	Localization
NM_001077499	Scn8a	No	43 (↑)	40 (↑)	Cell part	Binding molecular function	Localization
NM_026680	Golt1a	No	54 (up)	40 (↑)	Cell part	No	Localization

^∗^FCAbsolute: absolute ratio (no log scale) of normalized intensities between two groups. FCAbsolute value ≥ 40 was selected. ↑: upregulated; ↓: downregulated; No: not found in the database.

**Table 4:** The significant downregulated genes after NBXH-H treatment.

GenBank accession	Gene name	Groups			Gene ontology annotations		
		Water vs. NC	INH vs. Water	NBXH-H vs. Water	Cellular component	Molecular function	Biological process
NM_008206	H2-Oa	2 (↑)	4 (↓)	54 (↓)	Cell part	No	Cellular process
NM_175397	Sp110	4 (↑)	4 (↓)	50 (↓)	Intracellular part	Binding molecular function	Cellular metabolic process
NM_033324	Dgcr8	No	No	37 (↓)	Intracellular part	Binding molecular function	Metabolic process
NM_026605	Sympk	No	No	30 (↓)	Intracellular part	No	No
NM_008080	B4galnt1	No	2 (↓)	29 (↓)	Intracellular part	Catalytic activity	Metabolic process
NM_016670	Pknox1	3 (↑)	2 (↓)	29 (↓)	Intracellular part	Binding molecular function	Cellular metabolic process
NM_027425	Rufy2	No	4 (↓)	27 (↓)	Intracellular part	Binding molecular function	No
NM_027453	Btf3l4	2 (↑)	3 (↓)	26 (↓)	No	No	No
M22934	—	No	4 (↓)	25 (↓)	—	—	—
X56177	—	3 (↑)	7 (↓)	23 (↓)	—	—	—
U88691	—	2 (↑)	3 (↓)	22 (↓)	—	—	—
NM_009157	Map2k4	2 (↑)	No	22 (↓)	Intracellular part	Binding molecular function	Cellular metabolic process
NM_178688	Ablim1	4 (↑)	No	21 (↓)	Intracellular part	Binding molecular function	Cellular metabolic process
NM_138596	Med10	No	No	21 (↓)	Intracellular part	Transcription cofactor activity	Metabolic process
NM_176933	Dusp4	2 (↑)	No	21 (↓)	Intracellular part	Catalytic activity	Cellular metabolic process
NM_011513	Med22	No	No	21 (↓)	Intracellular part	Transcription cofactor activity	Metabolic process
NM_033078	Klrk1	No	No	20 (↓)	Cell part	Binding molecular function	Metabolic process
NM_026279	Bend5	No	No	20 (↓)	Intracellular part	No	No

**Table 5:** Significantly upregulated or downregulated signaling pathways associated with immunity and inflammation.

Pathway ID	Definition	Enrichment score			Annotation
		Water vs. NC	INH vs. Water	NBXH-H vs. Water	
mmu04668	TNF signaling pathway	+2.98463	-1.351648	+6.997055	Activated TNF binds to its receptors (TNFR1, TNFR2) resulting in the trimerization of TNFR1 or TNFR2. TNFR1 signaling induces activation of many genes, primarily controlled by two distinct pathways, NF-kappa B pathway and the MAPK cascade, or apoptosis and necroptosis. TNFR2 signaling activates NF-kappa B pathway including PI3K-dependent NF-kappa B pathway and JNK pathway leading to survival.
mmu04810	Regulation of actin cytoskeleton	-3.945801	-2.522185	+3.033837	Cytoskeleton proteins and their regulation proteins could be influenced seriously in *M. tuberculosis* infection host cells leading to the apoptosis of host cells.
mmu04014	Ras signaling pathway	-4.797349	+1.453685	+2.828953	The Ras proteins are GTPases that function as molecular switches for signaling pathways regulating cell proliferation, survival, growth, migration, differentiation, or cytoskeletal dynamism.
mmu04510	Focal adhesion	-7.79861	NS	+2.658141	Focal adhesions play essential roles in important biological processes. The expression of focal adhesion decreased, which led to a significant decrease in the regulation of extracellular matrix adhesion.
mmu04151	PI3K-Akt signaling pathway	-5.440354	-1.55789	+2.464812	PI3K catalyzes the production of PIP3 at the cell membrane to activate Akt. Once active, Akt can control key cellular processes by phosphorylating substrates involved in apoptosis, which will help the host to clear *M. tuberculosis*.
mmu04020	Calcium signaling pathway	-1.670006	+2.49438	+1.526486	*M. tuberculosis* infection leads to increased intracellular calcium influx or release of calcium ions from intracellular calcium pools to activate intracellular calcium signaling pathways, which will activate the expression of genes encoding intracellular anti-inflammatory-related protein and an immune protective related protein, enhance the killing and phagocytic capacity of immune cells such as macrophages, and ultimately clear *M. tuberculosis* in *vivo*.
mmu04010	MAPK signaling pathway	-2.125314	NS	+1.443387	The mitogen-activated protein kinase (MAPK) cascade is a highly conserved module that is involved in cell proliferation, differentiation, and migration. Activation of the MAPK signaling pathway promotes apoptosis, which facilitates host clearance of *M. tuberculosis* in infected cells.
mmu04350	TGF-beta signaling pathway	-1.700798	NS	+1.326878	The transforming growth factor-beta (TGF-beta) family members are structurally related secreted cytokines found in species ranging from worms and insects to mammals. A wide spectrum of cellular functions such as proliferation, apoptosis, differentiation, and migration are regulated by TGF-beta family members.
mmu04064	NF-kappa B signaling pathway	+1.875102	-1.565857	-1.38673	NF- kappa B signaling pathway plays an important role in the development of TB infection by participating in stress response and the regulation of transcription of genes related to immune cell activation, proliferation, differentiation, and apoptosis. *M. tuberculosis* infection causes abnormal activation of the NF-kappa B signaling pathway.
mmu04620	Toll-like receptor signaling pathway	+2.628034	-2.302575	-1.423933	*M. tuberculosis* is sensed by several pattern recognition receptors, including Toll-like receptor 2. TLR2 plays a prominent role in the induction of host immune responses during mycobacterial infection.
mmu04672	Intestinal immune network for IgA production	+2.266181	+1.371708	-2.595042	Secreted IgA promotes immune exclusion by entrapping dietary antigens and microorganisms in the mucus and functions for the neutralization of toxins and pathogenic microbes. NBXH extract immunotherapy can significantly reduce the overexpression of IgA induced by *M. tuberculosis* infection.
mmu04660	T cell receptor signaling pathway	+4.012943	NS	-4.361177	Activation of T lymphocytes is a key event for an efficient response of the immune system. T cell receptor (TCR) plays a key role in the function of T cells and the formation of immune synapses. It provides a connection between T cells and antigen-presenting cells. TB infection activates T cell, and NBXH treatment could regulate T cell activation negatively to keep it from overreacting.

NS: there were no significant differences in enrichment scores between the two groups.

## Data Availability

All data used to support the findings of this study are included within the article and the supplementary information files.

## Abbreviations

BP: Biological process
CC: Cellular component
CFUs: Colony-forming units
DE: Differentially expressed
DUSP4: Dual-specificity MAP kinase phosphatase 4
GO: Gene Ontology
INH: Isoniazid
KEGG: Kyoto Encyclopedia of Genes and Genomes
KLRK1: NK cell lectin-like receptor K1
MDR: Multidrug resistant
MF: Molecular function
MHC II: Major histocompatibility complex class II
NBXH-H: High-dose NBXH extracts
NBXH-L: Low-dose NBXH extracts
NBXH-M: Medium-dose NBXH extracts
NC: Normal control
NK: Natural killer
PBMCs: Peripheral blood mononuclear cells
SEM: Standard error of the mean
TB: Tuberculosis
TB-IRIS: Tuberculosis-associated immune reconstitution inflammatory syndrome
TCM: Traditional Chinese medicine
XDR: Extensively drug resistant.

## Supplementary Materials

Supplementary Materials Figure S1: GO BP, CC, and MF classification between before and after *M. tuberculosis* infection or NBXH-H treatment. The number of up- or downregulated DE genes in the top 10 terms of the biological process, cellular component, and molecular function classification was determined from the Water *vs.* NC group (A–F) or the NBXH-H *vs.* Water group (G–L). Table S1: differentially expressed genes (pass volcano plot). Table S2: significant up- or downregulated pathways.
