# Supplementary figures and images for: Chinese Traditional Medicine NiuBeiXiaoHe (NBXH) Extracts Have the Function of Antituberculosis and Immune Recovery in BALB/c Mice
Source: J Immunol Res. 2021 Jan 18;2021:6234560. doi: 10.1155/2021/6234560 (PMC7857905; doi:10.1155/2021/6234560)

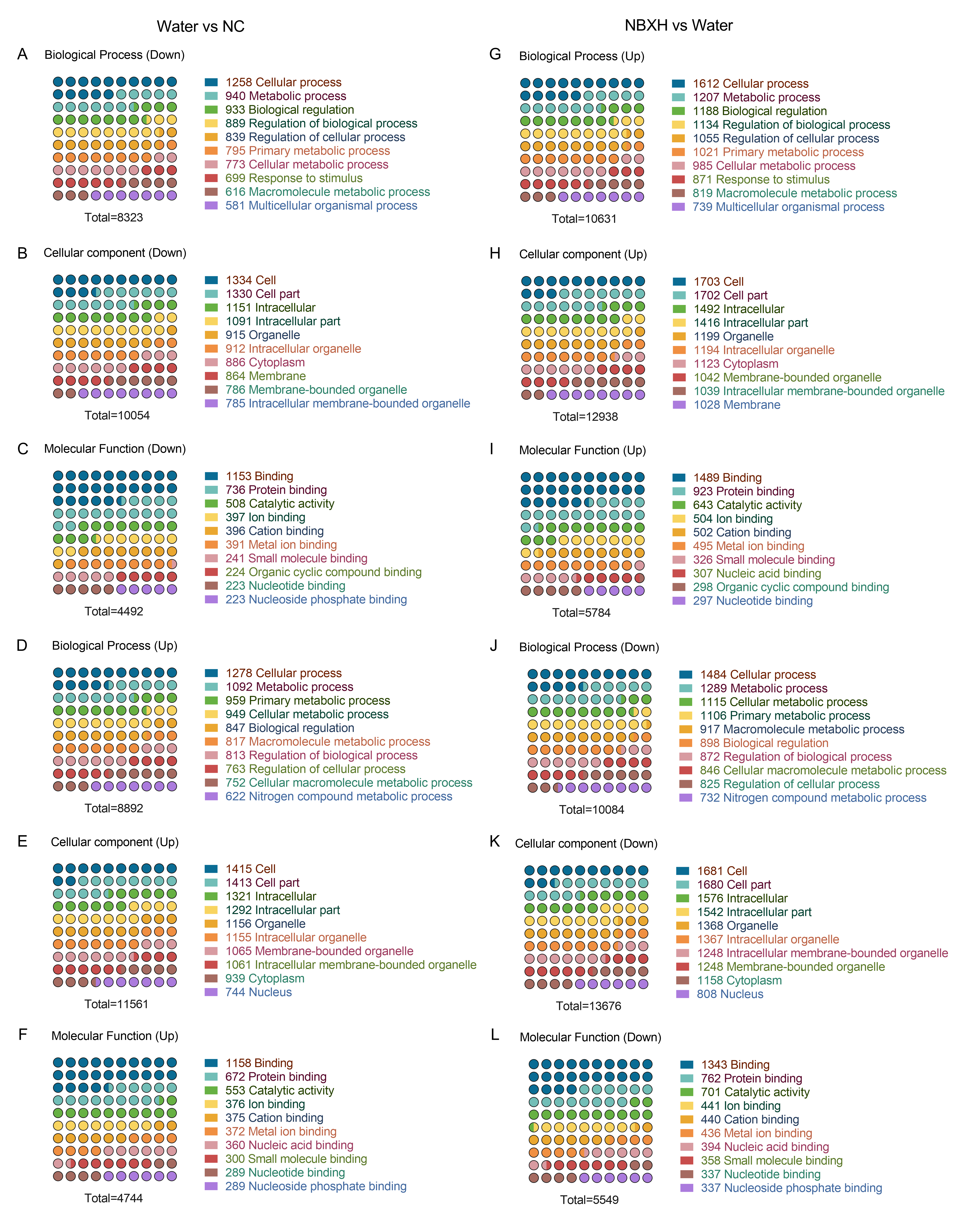

Supplement: Supplementary Materials — Figure S1: GO BP, CC, and MF classification between before and after M. tuberculosis infection or NBXH-H treatment. The number of up- or downregulated DE genes in the top 10 terms of the biological process, cellular component, and molecular function classification was determined from the Water vs. NC group (A–F) or the NBXH-H vs. Water group (G–L). Table S1: differentially expressed genes (pass volcano plot). Table S2: significant up- or downregulated pathways. [file 6234560.f1.zip › Figure S1-GO BP CC MF classification.tif]
